# Temporal Acoustic Emission Index for Damage Monitoring of RC Structures Subjected to Bidirectional Seismic Loadings

**DOI:** 10.3390/ma12172804

**Published:** 2019-08-30

**Authors:** Chihab Abarkane, Francisco J. Rescalvo, Jesús Donaire-Ávila, David Galé-Lamuela, Amadeo Benavent-Climent, Antolino Gallego Molina

**Affiliations:** 1 Building Engineering School, University of Granada, 18071 Granada, Spain; 2 Department of Mechanical and Mining Engineering, University of Jaén, 23009 Jaén, Spain; 3 Department of Mechanical Engineering, Universidad Politécnica de Madrid, 28040 Madrid, Spain

**Keywords:** acoustic emission, signal processing, concrete structure, damage, seismic loadings

## Abstract

This paper shows the acoustic emission (AE) analysis recorded during the loading process of reinforced concrete (RC) structures subjected to bidirectional seismic loadings. Two waffle plates (bidirectional) supported by isolated square columns were tested on a shaking table with a progressive and increasing ground acceleration until the final collapse. Each specimen was subjected to a different loading history. A relevant delay in the beginning of the significant AE energy is observed as the peak value of the ground acceleration increases. Based on this result, a new AE temporal damage index (TDI), defined as the time difference between the onset of the significant AE activity and the onset of the loading that causes this AE activity, is proposed and validated by comparing it with the plastic strain energy released by the concrete, typically used as a reliable damage level indicator. Good agreement was observed for both specimens and seismic inputs.

## 1. Introduction

In areas of moderate to high seismicity, earthquakes are by far the most challenging loading action for buildings and civil structures, due to their financial impact, concerning material losses, and especially to human impact, which often results in mortality [1]. The structures are typically designed for two levels of seismic intensity. Level I earthquakes are expected to occur once in several decades, and thus a building or civil construction can be subjected to several earthquakes of Level I during its life time. The structures are designed to endure Level I ground motions with minor damage. In the case of reinforced concrete (RC) structures, minor damage means admitting concrete cracking but small or no yielding of the reinforcing steel. Level II earthquakes are expected to occur once in several hundred years, and building or civil construction are designed to experience one earthquake of Level II without collapse, but admitting severe damage. This involves extensive concrete cracking/crushing and large plastic deformations in the reinforcing steel. Knowing the level of damage of the structure after earthquakes of both Level I or II is of prime importance to decide whether the structure needs to be repaired or not, and to what extent. As mentioned above, in the case of RC structures, the damage is mainly manifested through the concrete cracking/crushing, which, depending on its severity, can trigger other fatal deterioration mechanisms, such as, for example, the debonding between the concrete and the reinforcing steel. Assessing the severity of the damage on the concrete after an earthquake cannot be done by visual inspection mainly due to two reasons. One is that structural elements are covered by brick veneers, casing, cement plaster etc., that can hide the cracks. The second and most important reason is related to the nature of the loading imposed by earthquakes. Ground motions impose on the structural elements from several tens to thousands of cyclic deformations (not monotonic) and this causes cumulative damage. Cumulative damage cannot be assessed from the apparent damage (i.e., from the maximum displacement/deformation experienced by the structure, or from the width or the extension of the concrete cracks). The problem is further complicated by the fact that the seismic loading is mainly bidirectional (not unidirectional). The vertical and rotational components of the ground motions are usually negligible in conventional structures. The evaluation of the damage cumulated in concrete requires calculating the (cumulated) plastic strain energy dissipated by this material. However, the latter implies a cumbersome and expensive instrumentation of the structure with strain gages, accelerometers, displacement transducers, etc. This instrumentation can be done at the research level in structures tested in a laboratory, but it is not feasible in general in real conventional structures due to the economic cost.

An affordable alternative is the application of the acoustic emission (AE) technique [2,3,4,5] that has proven to be very useful for structural monitoring [6,7,8,9]. Structural health monitoring (SHM) is a process of diagnosis (i.e., detection, identification and assessment of flaws or conditions that affect or may affect the future safety or performance of the structure) and monitoring of the condition of the structures (i.e., follow the changes in the condition of the structure) normally performed during their operation. The AE method fits uniquely with these requirements, thus being considered as an excellent alternative for SHM. The temporal damage index (TDI) proposed in this study could be used in the SHM framework as follows. The structure would be monitored by means of several AE transducers located close to the potential or existing cracks, and by one or several accelerometers located close to the source of the dynamic loading (e.g., the foundation of the structure). The AE and the acceleration would be measured continuously or when a trigger level of acceleration is attained. The TDI index proposed in this study would be calculated at fixed or variable intervals of time, and the damage on the structure in terms of the accumulated plastic strain energy would be assessed on the basis of the good correlation with TDI index.

Recently, the authors used successfully the AE technique in structures subjected to uniaxial earthquakes reproduced by the shaking table of the University of Granada [10,11,12,13,14,15,16]. In particular, the correlation between the plastic energy dissipated by the concrete and the AE energy was proven to be a good strategy for verifying the reliability of the AE method in assessing the accumulated damage on concrete [11,14,15,16]. This work is a further step, through which the satisfactory results obtained for the structures tested under unidirectional seismic loadings are also obtained in the RC structure subjected to biaxial seismic loads. This further validates the reliability of the AE energy in realistic seismic scenarios.

An important element of the AE technique is the analysis of signals and its parameters. Many studies have been conducted on the classification of signals according to the source mechanism [17,18,19,20], some of which have focused on distinguishing those genuine signals inherent in the concrete cracking from signals generated by undesired noise or friction between the cracks [12,15,16]. During the works carried out by the Association of American Railroads [21] it was inferred that some secondary mechanisms like mechanical friction are characterized by the signals of long duration and low amplitude. This has resulted in the development of the Swansong filter technique [22], the variant of which was successfully used in this work. After the application of this filter, the evaluation of the acoustic energy emitted during each particular seismic event was analyzed. It was observed that the significant emission occurs after a particular delay with respect to the seismic event beginning. Moreover, this delay progressively increases as the earthquake’s intensity increases (its peak-ground acceleration), so it can be used as an index of damage, tentatively proposed and called as the temporal damage index (TDI) in this work.

## 2. Materials and Methods

### 2.1. Specimens and Experimental Set-up

**Figure 1:**
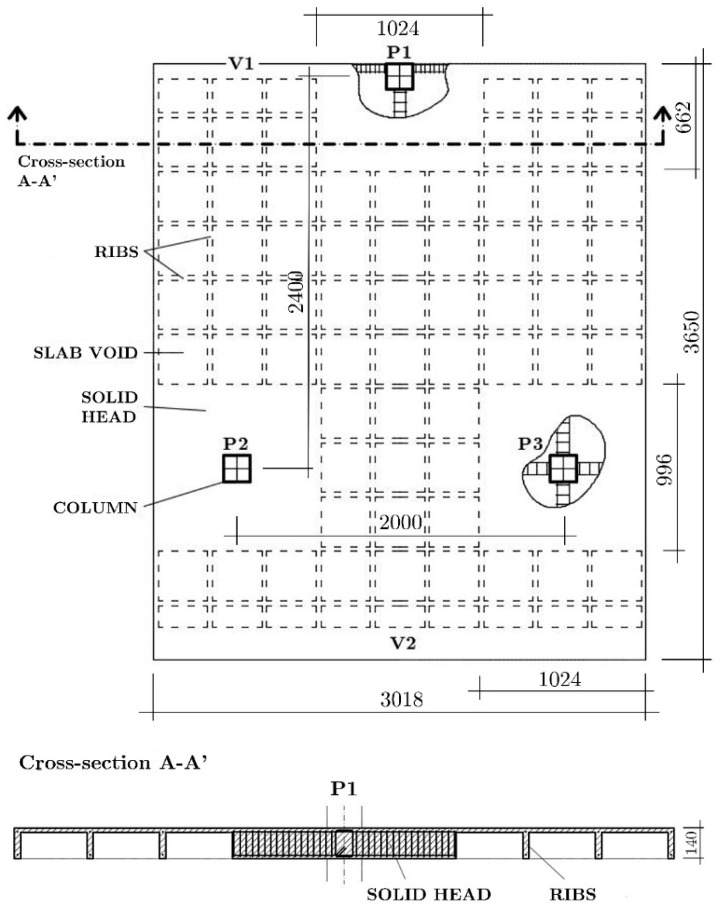
Floor plan and waffle plate section of BS1 and BS2 specimens. Dimensions in mm.

First, a prototype of the RC structure having three floors and constituted of waffle flat plates supported on isolated square columns was designed. The depth of the plate was 0.35 m and had a regular pattern of voids and a solid zone around the columns. The voids formed an orthogonal grid of ribs separated by 0.83 m. From this prototype, a sub-structure of one and a half floors and one and a half span was selected. The sub-structure was scaled by the factor *λ_d_* = 2/5 for the linear dimensions and *λ_σ_* = 1 for the stress to define the characteristics of the test specimens. The two identical test specimens, referred to hereafter as BS1 and BS2, were built in the Laboratory of Structures of the University of Granada. The tests specimens consisted of a waffle plate of 3.65 × 3.02 × 0.14 m^3^ supported on three columns of square Section 16 × 16 mm^2^, as shown in Figure 1. The dimensions of the solid zone around column P1 (see Figure 1) was 1.02 × 0.66 m^2^, and those around columns P2 and P3 (see Figure 1) were 1.02 × 1.02 m^2^. The connection of column P1 with the plate will be called hereafter, exterior connection, and the connection of column P2 or P3 with the plate, interior connections. Further details about the specimens and their elaboration can be found in [23].

**Figure 2:**
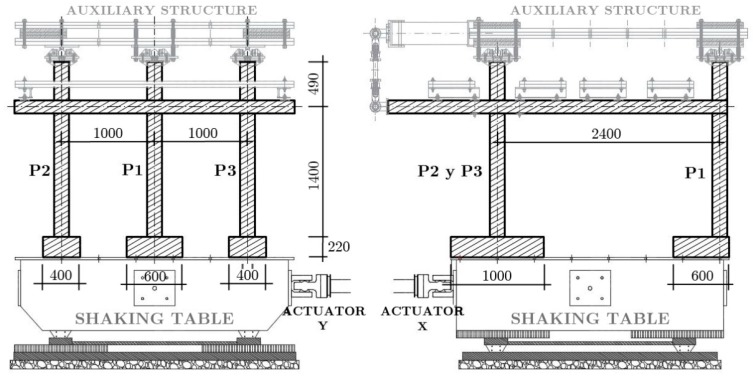
Elevations of the specimens installed on the shaking table. Dimensions in mm.

**Figure 3:**
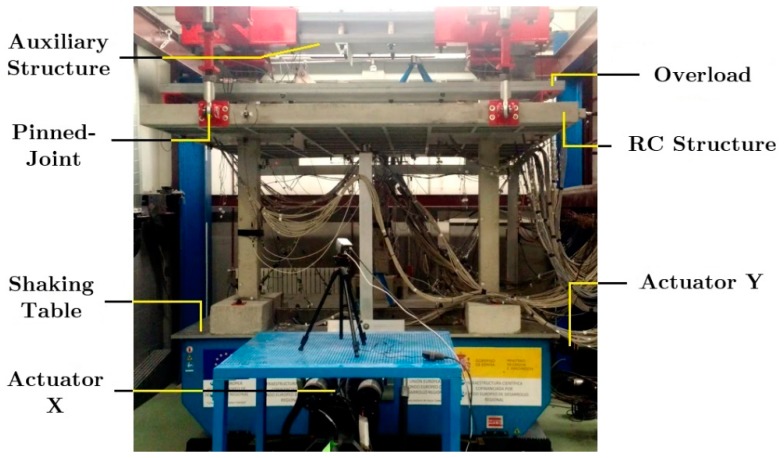
General setup of the seismic test: Shaking table of the Laboratory of Structures of the University of Granada (actuators X and Y), reinforced concrete (RC) main structure with overload, and auxiliary metallic structure.

The specimens were placed on the shaking table of the Laboratory of Structures of the University of Granada as shown in Figure 2 and Figure 3, and tested under dynamic loadings. One additional scaling factor of *λ_a_* = 1 for the acceleration was used for the dynamic tests. In order to simulate the boundary conditions of the sub-structure within the prototype, bi-articulated steel bars and pinned-joints were installed at the free ends of the upper columns and on the plate. The additional steel blocks (overloads) were attached at the top of the plate and at the top half of columns of the second story to represent the gravity loads acting on the floors and to satisfy similitude requirements.

**Figure 4:**
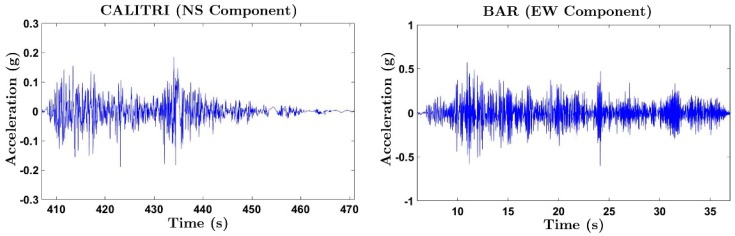
Original accelerogram of the earthquake recorded in the seismic station. Left: Calitri. Right: Bar-Skupstina Opstine.

Specimen BS1 was subjected to the two horizontal components of the ground motion recorded at Calitri station during the Campano-Luchano earthquake (Italy, 1980). Specimen BS2 was subjected to the two horizontal components of the ground motion recorded at Bar-Skupstina Opstine station during the Montenegro earthquake (1979). The ground accelerograms corresponding to the component with the highest peak-ground acceleration (PGA) for each earthquake are shown in Figure 4. The first earthquake (Calitri) is a far-field ground motion without pulses. The second ground motion (Bar) is an impulsive near-fault earthquake. Each specimen was subjected to successive seismic tests, referred to as seismic simulations hereafter. In each seismic simulation, the two components, north-south and east-west, of the ground motion where applied simultaneously by actuators X and Y, respectively (see Figure 3). In each seismic simulation, the accelerograms were scaled in amplitude to increasing values until the collapse of the specimen. Table 1 shows the PGA applied to the shake table in each seismic simulation expressed in terms of the acceleration of gravity (g). The name of each seismic simulation in Table 1 (C35, C100 etc. for specimen BS1, or B15, B5 etc. for specimen BS2) was formed with the first letter of the name of the acceleration record, and a number that indicated the percentage of the original PGA applied to the shake table during the seismic simulation. Thus, C200 for example, identifies a seismic simulation with Calitri record whose peak acceleration is 2 times that of the original (unscaled) record. Note that in the simulations, C200 and C200b in the case of specimen BS1, and in simulations B130 and B130b in case of specimen BS2, the peak acceleration was kept the same.

### 2.2. AE Monitoring

**Figure 5:**
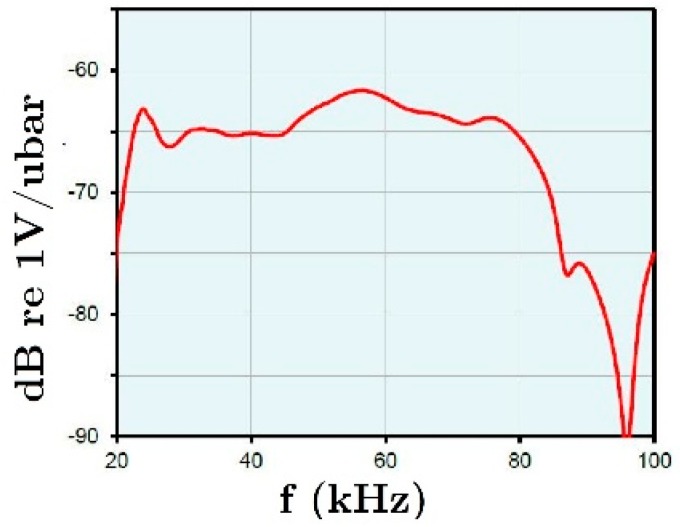
Distribution of the acoustic emission (AE) sensors on the specimens.

Furthermore, 18 sensors were placed on the specimens to capture the acoustic emission (AE) released by the specimen during the seismic tests. Six sensors (labelled 10–15 in Figure 5) were placed on the exterior solid head, seven sensors (labelled 1–7 in Figure 5) on the interior solid head of column P2, two sensors at the bases of columns P1 and P2, and three at the pinned-joints at the top end of the columns. The VS30-V broadband low-frequency sensors with 34 dB_AE_ gain preamplifiers were used for all channels. The AE signals captured by the sensors were recorded with an AMSY-5 acquisition equipment (Vallen System, Icking, Germany). The acquisition threshold was setup to 45 dBAE and a passing filter from 30 kHz to 120 kHz was setup in order to avoid mechanical and electromagnetic noise as much as possible. Other actions at the instrumentation level were conducted in order to prevent the electromagnetic noise.

**Figure 6:**
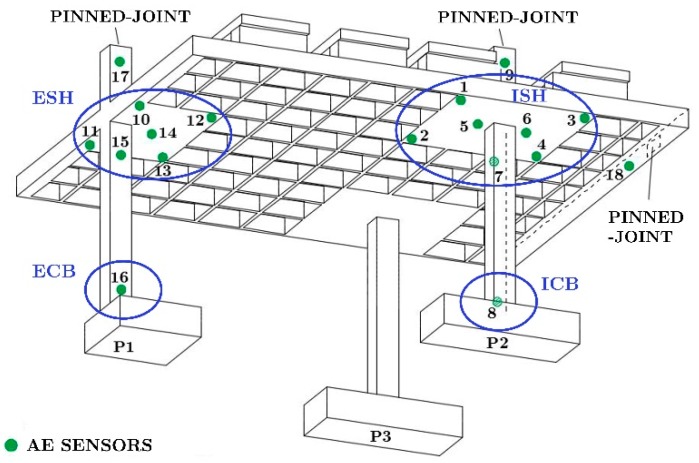
Sensitivity curve of sensor VS30-V.

The VS30-V sensors were manufactured by Vallen System (Icking, Germany). This type of sensor exhibits high and uniform response in the low frequency range (i.e., 25–80 kHz). Figure 6 shows the sensitivity curve of this type of sensor. The preamplifiers (model AEP4) and the acquisition system (AMSY-5) were manufactured also by Vallen System. The acquisition system, AMSY-5, has 18 channels for the AE sensors and two additional parametric entries that were used to record the shake table accelerations in the X and Y directions. Further details can be found in [24].

### 2.3. Plastic Strain Energy

During the test, the lateral displacements between the plate and the shaking table were measured with 8 displacement transducers (LVDT sensors, (RDP Electronics, Wolverhampton, UK), the accelerations of the plate with 8 seismic accelerometers and the plastic deformation of the steel reinforcements with 472 linear strain gauges attached to the bars. The gauges were located in the regions of expected plastic deformations (i.e., the regions of maximum bending moments), that is, at the column ends and at the solid zone around the columns.

#### 2.3.1. Estimation of the Energy Absorbed by Columns

**Figure 7:**
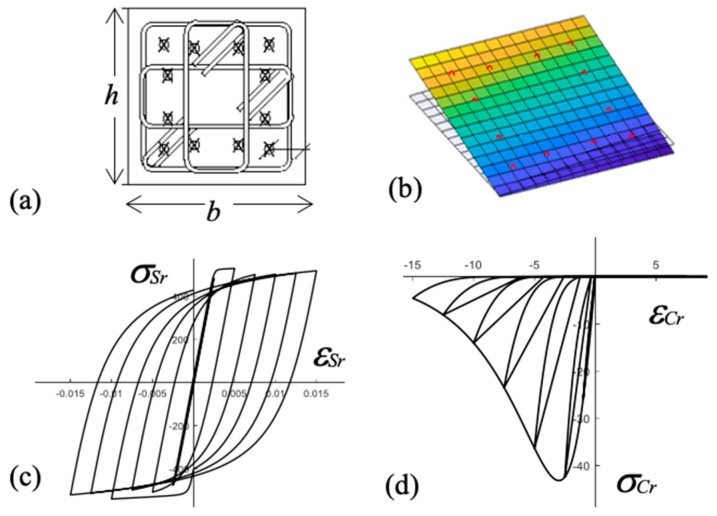
Column P1: (**a**) base cross section; (**b**) discretization of the cross section in fibers; (**c**) constitutive model for steel; (**d**) constitutive model for concrete.

Plastic deformations occurred at both ends of each column. The central part remained basically elastic (i.e., without cracks). The region of the plastic deformations at each end of the column is commonly referred to as plastic hinge and it is delimited by two parallel planes perpendicular to the column axis spaced a distance $l_{p}$ measured from the point where the column axis contacts the plate. Past studies showed that $l_{p}$ can be taken equal to the depth of the column. The elastic strain energy stored and the plastic strain energy dissipated in a given plastic hinge k from instant *t* = 0 to $t=t_{t}$, $W_{PH,k}$, is the sum of the energy stored/dissipated by concrete, $W_{C,k}$, and the energy stored/dissipated by steel rebars, $W_{S,k}$. In this study $W_{C,k}$ and $W_{S,k}$ are estimated as follows. Figure 7a shows the typical cross section of a column at the plastic hinge region. The rebars instrumented with strain gauges are indicated with the symbol ×. The cross section of the plastic hinge, of depth h and width b, is divided in N × N fibers as shown in Figure 7b, where N = 14. The depth of each fiber is h/N and the width b/N. A different material (steel or concrete) is assigned to each fiber, depending on the position of the rebars in the RC section. As usual in RC members subjected to flexural deformations, it is assumed that plane sections remain plane after bending. This is commonly called in the literature, the plane remaining plane assumption and it is shown in Figure 7b, where the colored part represents the cross section after bending and the part without coloring, the cross section before bending. Based on this assumption, the strain at any instant t, $\varepsilon_{sr}(t)$, of any rebar r of the cross section of the plastic hinge is obtained from the strains measured by the gauges adhered to the longitudinal reinforcement by plane interpolation. The stress $\sigma_{sr}(t)$ associated with $\varepsilon_{sr}(t)$ is estimated using the Pinto-Menegoto [23] constitutive model for steel shown in Figure 7c. Naming $A_{sr}$ the area of a rebar *r* and assuming that plastification develops uniformly in the length $l_{p}$, $W_{S,k}$ at a given instant t is calculated as follows:

(1) $$W_{S,k}=\overset{R}{\underset{r=1}{\sum }}\int_{\varepsilon_{sr}\left(0\right)}^{\varepsilon_{sr}\left(t_{t}\right)}l_{p}A_{sr}\sigma_{sr}d\varepsilon_{sr}$$

Here, *R* is the number of longitudinal rebars of length $l_{p}$ in the plastic hinge. On the other hand, using the plane remaining plane assumption and assuming also that there is no slippage between the longitudinal rebars and the surrounding concrete, the strain $\varepsilon_{Cj}(t)$ at a given concrete fiber *j* of the cross section is calculated from the strains $\varepsilon_{sr}(t)$ provided by the longitudinal rebars through the plane interpolation (Figure 7b). The corresponding stress in fiber *j*, $\sigma_{Cj}(t)$, is estimated from $\varepsilon_{Cj}(t)$ using the constitutive model for concrete proposed by Maekawa [25] and shown in Figure 7d. Finally, assuming that plastification develops uniformly in the length $l_{p}$, $W_{C,k}$ at a given instant *t* is given by:

(2) $$W_{C,k}=\overset{}{\underset{concrete\;fibers}{\sum }}\int_{\varepsilon_{sr}\left(0\right)}^{\varepsilon_{sr}\left(t_{t}\right)}l_{p}\frac{b}{N}\frac{h}{N}\sigma_{Cj}d\varepsilon_{Cj}$$

where the summation extends to all concrete fibers of the cross section. The elastic and plastic strain energy stored/dissipated by a given plastic hinge *k* is thus:

(3) $$W_{PH,k}=W_{S,k}+W_{C,k}$$

#### 2.3.2. Estimation of the Energy Absorbed by the Waffle-Flat Plate

Most of the energy absorbed by the plate was dissipated at the exterior plate-column connection, and more precisely, around the region where column P1 intersected the solid zone. This caused severe damage as shown later in Figure 11a,d that shows the state of the exterior connection at the end of the tests.

The rest of the plate remained basically elastic (i.e., the energy dissipated through plastic deformations was negligible), and only minor cracks were observed on the solid parts of the plate around the columns. The energy absorbed by the plate cannot be evaluated directly as in the case of the columns, because the number of strain gauges was limited and the geometry of the region where plastic deformations occur cannot be clearly defined, i.e., the concept of the plastic hinge of length *l_p_* developed for columns is not directly applicable to bi-dimensional plate elements. Nevertheless, a rough estimation can be made indirectly as the difference between the total energy absorbed by the whole structure, *E_I_−W_k_−W_ξ_,* and the energy absorbed by the columns. Here *E_I_* is the total energy input by the earthquake, *W_k_* is the kinetic energy and *W_ξ_* is the energy dissipated by the inherent damping of the structure. *E_I_, W_k_* and *W_ξ_* can be estimated from the measurements provided by the accelerometers and the displacement transducers. A detailed explanation of how this calculation is done can be found in reference [23].

## 3. Results

A zonal analysis of the AE and damage patterns was carried out. In particular, four zones of the specimens was considered separately: a) ISH area (solid head of the interior connection—column P2), for which the sensors 1, 2, 3, 4, 5, 6 and 7 were setup as normal ones and the rest as guard; b) ESH area (solid head of the exterior connection—column P2), for which the sensors 10, 11, 12, 13, 14, and 15 were setup as normal ones and the rest as guard.; c) Base of interior column P2 (ICB); d) the base of the exterior column P1 (ECB) (see Figure 6). These zones were selected because they are the regions of maximum bending moments under lateral earthquake loadings, and thus, the regions where potential plastic deformations can develop.

**Figure 8:**
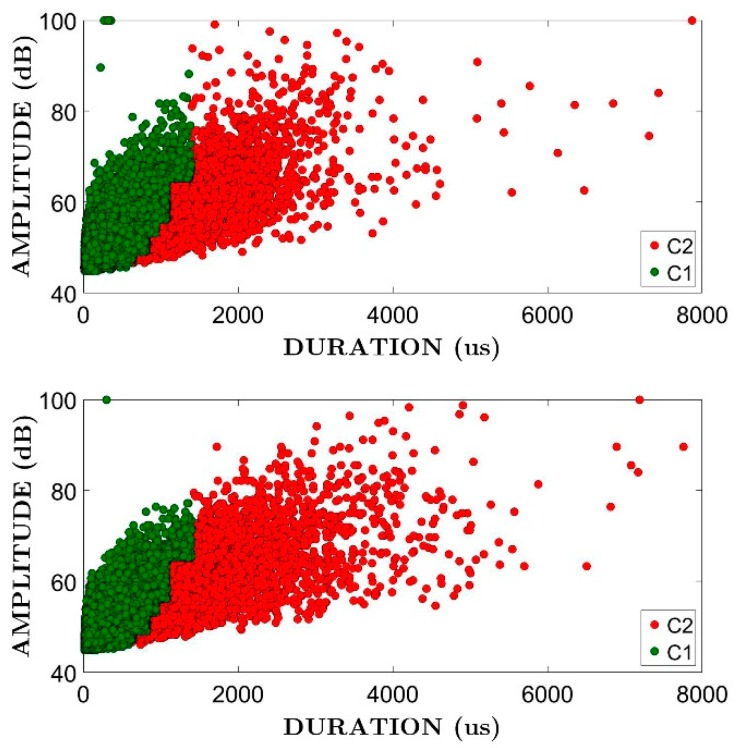
Amplitude-Duration plots for AE signals. Zone solid head of the exterior connection (ESH) of specimens BS1 (**top**) and BS2 (**bottom**). Green: passing the Swansong filter: Red: No passing the Swansong filter.

Firstly, the AE signals were filtered by a Swansong filter in order to avoid those of long duration and low amplitude, usually coming from the secondary mechanisms as mechanical friction. Table 2 lists the limit values previously proposed by Soltangharaei et al. [22] for the RC structures with dimensions comparable to BS1 and BS2 for the Swansong filter. Basically, the filter eliminates the AE signals with a duration higher than DL, with DL depending on its amplitude as shown in Table 2. As an example, Figure 8 shows the AE signals passing the filter (in green) and those not passing the filter (in red), for the whole seismic simulations in a particular area of both BS1 and BS2 specimens. It was noted that 100 dBAE is the maximum amplitude range of the measuring system. No passing signals (filtered out) were 11%, 11%, 4% and 3% for IHS, EHS, ICB and ECB zones, respectively for specimen BS1, and were 13%, 15%, 6% and 4% for IHS, EHS, ICB and ECB zones, respectively for specimen BS2.

### 3.1. Accumulated AE Energy

In the type of structures investigated in this study (waffle flat-plate RC systems), the exterior connection is typically the weakest part, whether the seismic loading is unidirectional [26] or bidirectional [23,27]. In the latter case, the damage concentration in the exterior connection is enhanced by the torsion of the plate induced by the bidirectional effects.

**Figure 9:**
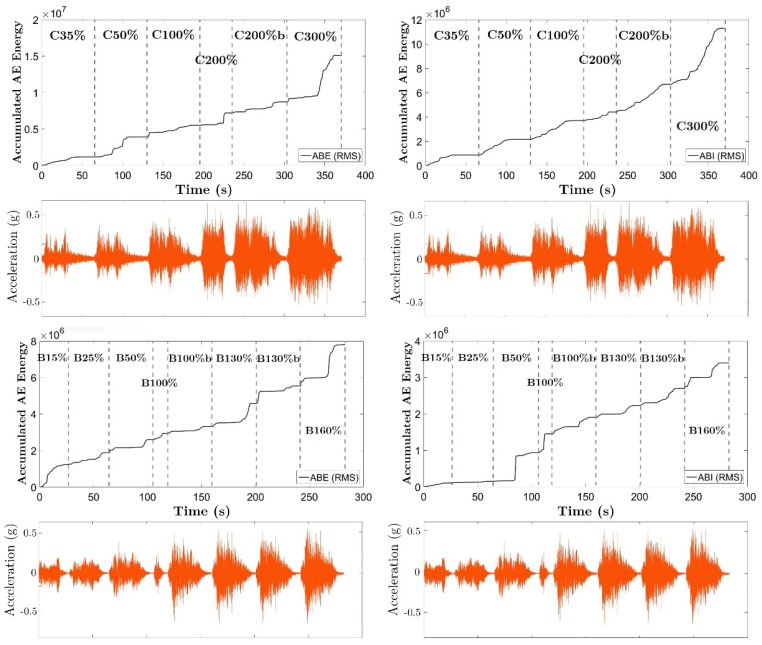
The accumulated AE energy in the ESH and ISH zones for BS1 (top) and BS2 (bottom) specimens. Bottom of the figures: Ground acceleration as a function of time.

Figure 9 shows the history of accumulated AE energy released in the ESH and ISH zones for each specimen. Below each history of the accumulated AE energy, the history of the acceleration applied to the shake table in one of the directions (the one of maximum PGA) were also plotted.

The plots in Figure 9 reveal a greater accumulation of AE energy in the exterior connection—column P1 (ESH area)—compared to the interior connection—column P2 (ISH). The difference is even greater if it is taken into account that the volume of the solid zone of the slab in the exterior connection (ESH) is smaller than that of the interior connection (ISH). Further, a clear increasing of the released AE is observed as the acceleration applied to the shake table increases.

**Figure 10:**
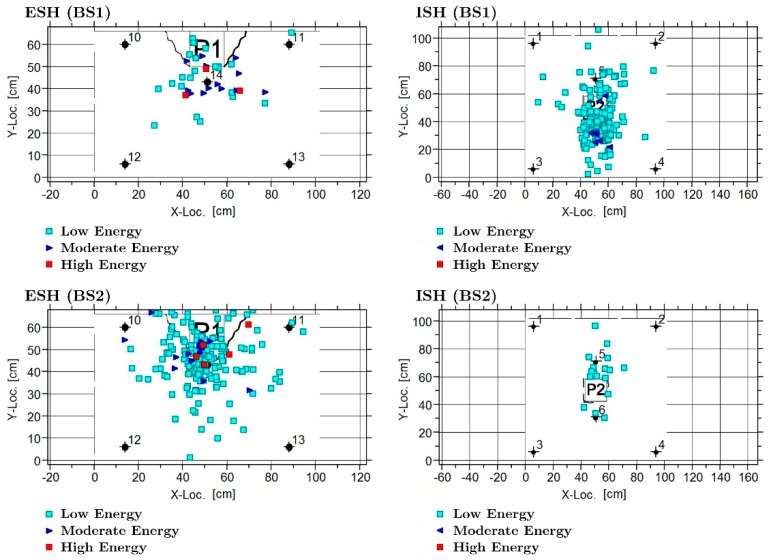
Planar location of the AE events at ESH and ISH zones for specimens BS1 and BS2.

**Figure 11:**
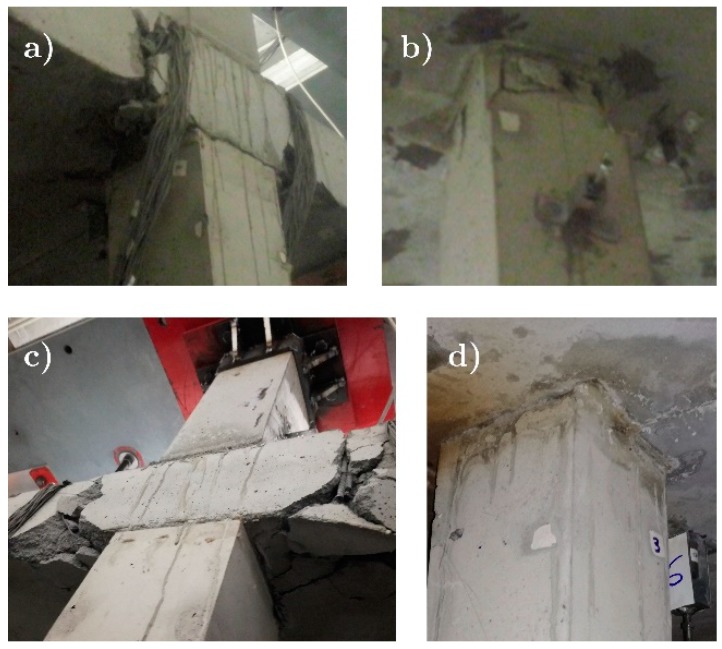
Final damage at the column-plate connections: a) ESH (BS1), b) ISH (BS1), c) ESH (BS2) and d) ISH (BS2).

Figure 10 shows the results of the AE planar location (2D) analysis performed in both the interior (ISH) and exterior (ESH) connections. A clear concentration of events can be observed around the top of the columns of the first story in both cases. The localized events were tentatively classified in three intensity levels: Low energy (light blue), moderate energy (blue) and high energy (red). It can be observed that some events of high energy were located at the exterior connection. In contrast, weaker events were recorded in the interior connection. The post-test visual inspection confirmed this result, since large cracks were observed in ESH and only small detachments in ISH. In both cases, damage occurred in the top of the column (see Figure 11).

### 3.2. Temporal Damage Index (TDI)

**Figure 12:**
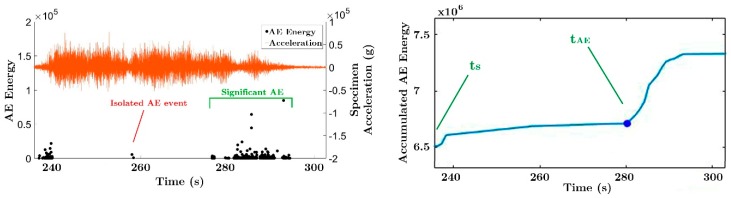
AE_SIGN_ (left) and the accumulated AE energy (right) during the C200b. ECB, specimen BS1.

**Figure 13:**
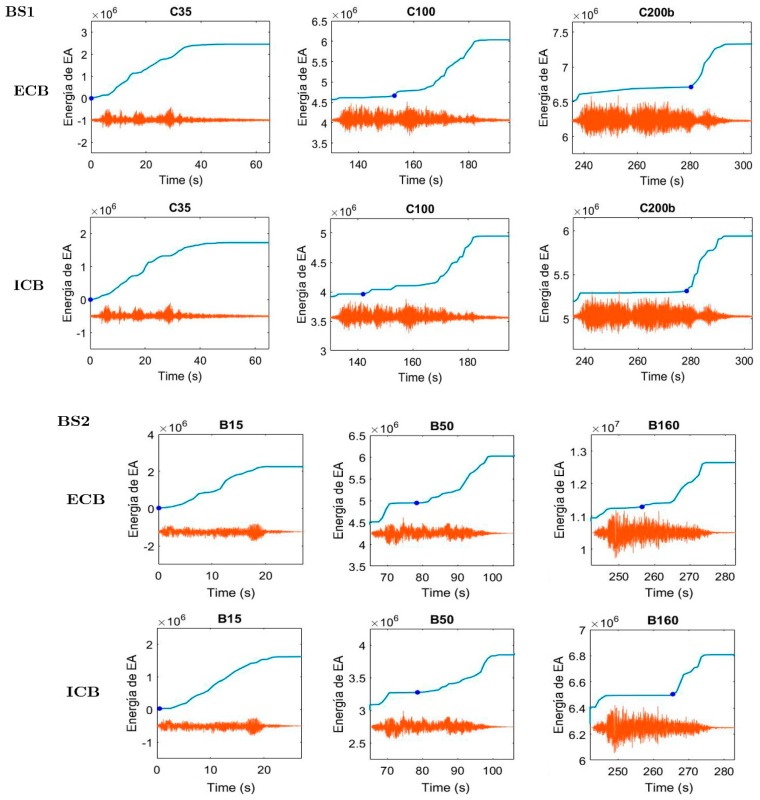
The accumulated AE energy on 3 different seismic simulations with the beginning of the *AE_SIGN_*, *t_AE_*.

A deeper analysis of the AE energy of the events for each seismic simulation, as represented in Figure 12 and demonstrated in plots of Figure 13, shows that the significant acoustic emission (AE_SIGN_) arrives with a relevant delay with respect to the beginning of the seismic simulation. Moreover, this delay increases as the damage of the specimen increases, i.e., along the successive seismic simulations. The AE_SIGN_ for a particular seismic simulation is defined as an uninterrupted succession of AE events, without considering the previous isolated events (Figure 12). This behavior was observed for all the seismic simulations in both specimens. The TDI phenomenon can be attributed to several sources and to fully identify and understand all of them, additional studies are needed. One reason that can explain the delay of the AE energy onset as the acceleration increases is that in the first cycles, the steel receives the load dissipating plastic strain energy without much AE. After many cycles, different degradation phenomena happen (one of them being the deterioration of bond conditions between the steel and surrounding concrete) and the concrete starts to further crack.

Based on this relationship between the delay of the AE_SIGN_ and the peak-ground acceleration applied to the shake table (increasing peak-ground accelerations at the base correspond to increasing damage on the structure), a temporal damage index (TDI) is defined as the difference between the beginning of the seismic simulation ($t_{s}$) and the beginning of the AE_SIGN_, $t_{AE}$, i.e.:

(4) $$TDI=t_{AE}-t_{s}$$

**Figure 14:**
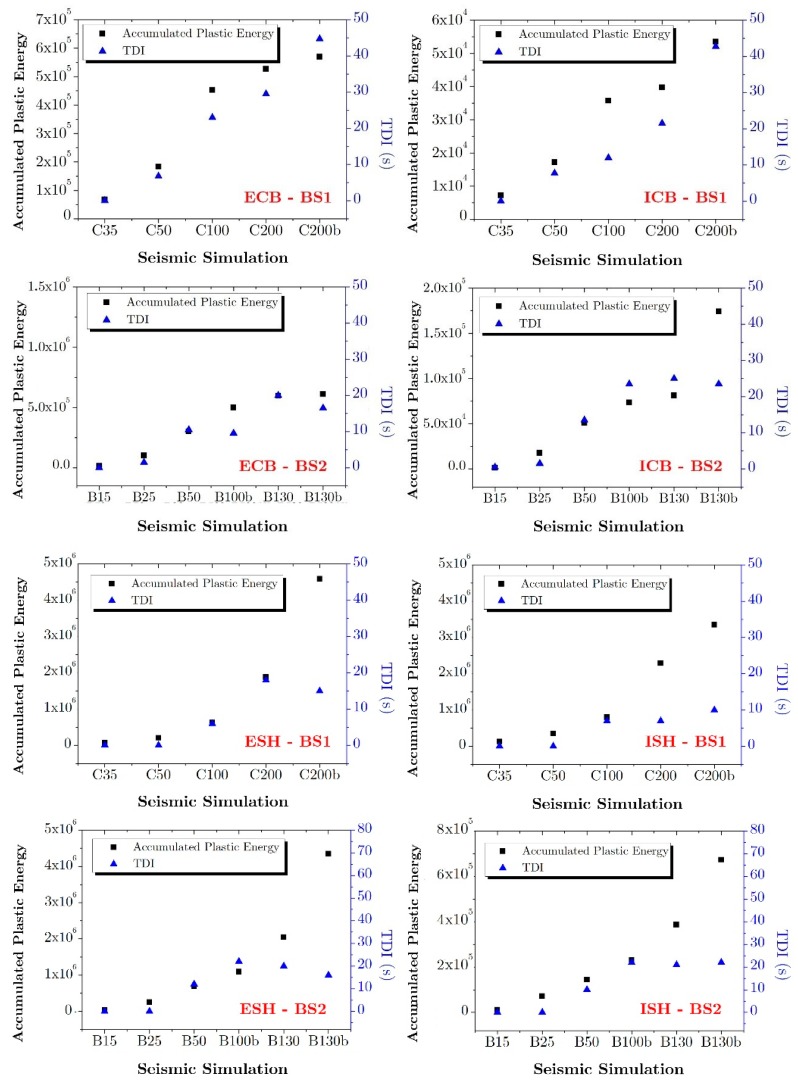
Correlation between the accumulated plastic energy $W_{C,p}$ and the temporal damage index (TDI).

Figure 14 compares the TDI index with the plastic strain energy released by the concrete accumulated at the end of each simulation, for zones ESH, ISH, ECB and ICB from both specimens, where the onset of the significant AE (TDI definition) has been picked by eye examination. In general, there is a progressive growing of both damage parameters as the peak acceleration increases. The agreement between both damage parameters is particularly good at the base of columns, where the amount of absorbed energy is directly and more reliably estimated as explained in Section 2.3.1. However, in the solid head connections, such agreement is only good up to simulations C100 and B100b, for which the most energy is dissipated by the columns. Beyond simulations C200 and B130, the amount of energy dissipated by the solid head is significant in comparison with the energy absorbed by the columns, and the fact that the energy dissipated by the plate is indirectly and roughly evaluated (see Section 2.3.2) could explain the worse agreement.

## 4. Conclusions

Shaking table tests were conducted on two scaled structures consisting of RC waffle-flat plates supported on isolated columns. Each test specimen was subjected to several seismic simulations of increasing intensity until they reached a severe level of damage. The two horizontal components of two different ground motions were applied simultaneously to reproduce a real seismic scenario. The test specimens were instrumented with strain gauges, accelerometers, displacement transduces and acoustic emission sensors, that allowed the damage to be monitored on the specimens and quantify it in terms of the cumulated plastic strain energy and AE energy. The results of the tests evidence the validity of AE energy for estimating the damage in concrete. The spatial concentration of AE energy in the solid head connection is in agreement with the observed damage. Based on the analysis of the history of AE energy during the successive seismic simulations, a new temporal index of damage (TDI) was proposed and compared with the damage experienced by the test specimens (expressed in terms of cumulate plastic strain energy) at the base of the columns and at the exterior and interior solid head connections. Good agreement was found between the TDI index and the energy dissipated at the base of the columns. The agreement is also satisfactory in the initial seismic simulations at the interior and exterior connections, but worsen in the last seismic simulations. The latter is probably due to the fact that in the last simulations, the plastic strain energy dissipated by the plate is large in comparison with that dissipated by the columns, and the energy dissipated by the solid head can be estimated only roughly and indirectly.

## Figures and Tables

**Table 1:** The peak-ground acceleration of each seismic simulation.

	**Specimen BS1**					
	**C35**	**C50**	**C100**	**C200**	**C200b**	
**PGA (g)**	0.07	0.10	0.19	0.42	0.42	
	**Specimen BS2**					
	**B15**	**B25**	**B50**	**B100**	**B130**	**B130b**
**PGA (g)**	0.06	0.09	0.19	0.37	0.48	0.48

**Table 2:** Duration limits (DL) for the Swansong filter applied to AE signals, depending on its amplitude. The values proposed by [22].

Amplitude (dB_AE_)	DL (μs)
41–43	>400
44–45	>500
46–47	>600
48–49	>650
50–53	>820
54–56	>940
57–65	>1080
66–100	>1400
